# Fabrication of crosslinker free hydrogels with diverse properties: An interplay of multiscale physical forces within polymer matrix

**DOI:** 10.1016/j.isci.2024.111227

**Published:** 2024-10-22

**Authors:** Tithi Basu, Debasish Goswami, Saptarshi Majumdar

**Affiliations:** 1 Department of Chemical Engineering, Indian Institute of Technology, Hyderabad 502285, Telangana, India

**Keywords:** Materials science, Polymers

## Abstract

Physical/chemical crosslinking and surface-modifications of hydrogels have been extensively endorsed to enhance their biomaterial functionalities. The latter approaches involve using toxic crosslinkers or chemical modifications of the biopolymers, limiting the clinical translation of hydrogels beyond short-term promising results. The current study aims to tailor the polymer’s structure to obtain customized applications using the same FDA-approved ingredients. PEGs of different molecular weights have been used to tune the van der Waal’s forces, NaCl has been used to alter the electrostatic interactions of the charged polymers, and glycerol has been used to tweak the H-bonding. Same crosslinker-free sodium alginate/gelatin hydrogel formulation unfolds multiple properties: controlled-release, self-healing, mesh size, storage modulus, degradation rate. The hydrogels, lacking in one aspect, displayed superior performance in another. This study, including experiments and molecular simulations, illustrates that developing new materials may not always be necessary, as the same polymeric matrix can generate immense variations in different aspects.

## Highlights

• Sodium alginate and gelatin hydrogels prepared without any chemical modifications
• Crosslinker free hydrogel engineered to provide multiple properties of the gel
• Similar formulation exhibits substantial changes in the macroscopic properties
• Controlled release, rheological properties tuned via physical forces of polymers

## Graphical abstract

**Figure:**
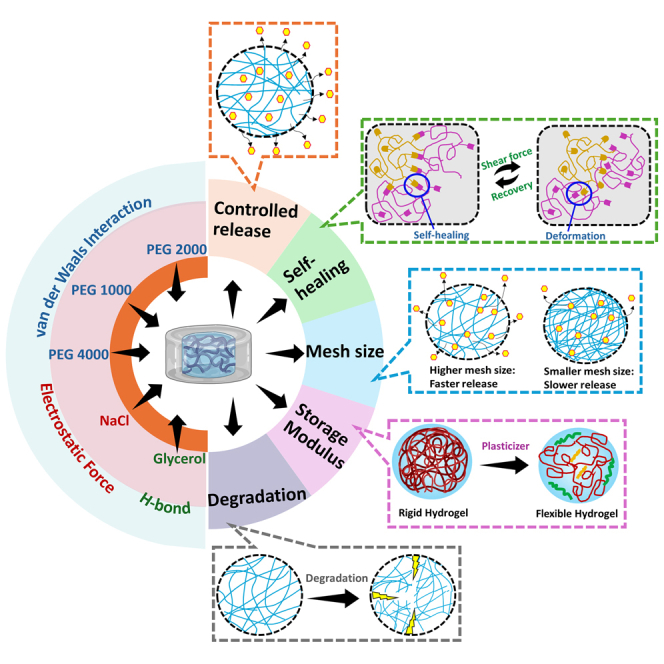

## Introduction

The functionality of hydrogel has been tremendously improved over the last decade by using surface modification, chemical crosslinkers, and a plethora of innovative techniques to enhance the performance of the gel. The field of hydrogels has received remarkable advances in the recent past owing to their multiple functionalities in oral drug delivery, scaffolds for tissue engineering, wound healing, eye drop formulation, medical implants, contact lenses, models for biological studies, shape memory, self-healing, and many others.^1^^,^^2^^,^^3^^,^^4^^,^^5^^,^^6^^,^^7^

Hydrogels are prepared using natural biopolymers to meet the biomaterial design requirements because of their excellent biocompatibility and biodegradability. However, these hydrogels are too soft or fragile and are prone to early degradation. Hence, several strategies have been developed to overcome the shortcomings of previous alternatives. The low mechanical strength and early degradation of biopolymeric hydrogel have been improved by using nanocomposite hydrogel, double network hydrogel, coordination interaction, host-guest interaction, microcrystals, covalent crosslinking, and chemical crosslinkers.^8^^,^^9^^,^^10^^,^^11^ Researchers have designed self-healing material using dynamic non-covalent bonds that break and reform during stretching, leading to the unfolding of polymer chains.^12^ In another research, poly(ethylene glycol) (PEG) and poly(vinyl alcohol) were crosslinked using borate ester and glycol chitosan by imine to fabricate a self-healing hydrogel with a double-dynamic network structure.^13^ However, there are drawbacks to these; for example, a higher chemical crosslinker content will stiffen the hydrogel, making it brittle and rigid.^14^^,^^15^ The potential of these techniques is promising, yet their clinical translation faces substantial challenges due to the presence of harmful chemical crosslinkers. The addition of different crosslinkers or chemical modification of the biopolymers may trigger side effects, which is concerning. Moreover, biocompatibility and biodegradability are essential for successful clinical translation of biomaterials. Biocompatibility studies using animal models can provide promising outputs immediately and in a quick time frame. However, the same is not assured in the long run. Thus, long-term compatibility is beyond the periphery of short-term *in vivo* studies, which is an issue of concern. Furthermore, the scale-up of the reported materials is complex because of their intricate composition and multiple preparation steps, leading to increased cost and time.^16^ Most *in vitro* models have low throughput and high cost; hence, new development of *in vitro* models is required.^17^ Thus, a more simplified approach is the need of the hour, which can deliver the same quality of release without compromising its efficacy and eliminate potential toxicity. To address these challenges, our research explores the idea of presenting different hydrogel properties using the same FDA-approved raw materials without any chemical modifications. This work is quite an upstream attempt as opposed to the regular trends of surface functionalization and/or chemical crosslinking to tune the hydrogel properties for customized applications. Fabrication of hydrogels without chemical crosslinkers can improve the safety of hydrogels in the long run.^18^ This work explores the in-depth polymeric interaction, taking sodium alginate and gelatin hydrogel as the primary, well proven resources. However, engineering a crosslinker-free or physically crosslinked gel is much more challenging. It requires understanding polymer chains in a solution and polymer entanglement in a gel, providing elasticity on a short timescale and viscosity on a long timescale.^19^ Physical crosslinking, like chain entanglement of polymer strands, can make the interactions dynamic and reversible within the hydrogel network.^20^^,^^21^ Due to their highly tunable properties, charged polymers have been thoroughly used in designing hydrogels. Kim et al. have shown that polymer arrangement can give rise to different properties. For example, a highly entangled polymeric network outnumbers the crosslinks of the hydrogel network, thus increasing the strength of hydrogel.^22^

Recent studies have shown the preparation of sodium alginate hydrogel using ellagic acid and PVA for wound dressing.^23^ Sodium alginate and gelatin are FDA-approved biopolymers, which are relatively cheaper and have minimal cytotoxicity. However, they lack mechanical strength and are subject to uncontrollable degradation. Genipin has been used to improve the mechanical properties of gelatin because of its low cytotoxicity; however, it is costly.^24^ Sun et al. have prepared self-healing injectable hydrogel using chitosan and sodium alginate, which contains dynamic covalent bonds.^25^ Dual crosslinked (ionic and covalent) sodium alginate hydrogel has also been designed for wound healing.^26^ Sodium alginate/gelatin (SA/G) hydrogel has shown improved hydrogel properties when modified with tannic acid.^27^ Physically crosslinked SA/G hydrogel has also been prepared; however, tuning the polymeric network of hydrogel to portray different properties was not discussed in any of the previous research.^28^^,^^29^ Research has been carried out to analyze the toughness properties of the sodium alginate/gelatin hydrogels, however, the polymeric interaction toward controlled release behavior, self-healing, and other properties were not shown.^30^ Much research has been conducted based on ionic crosslinking of alginate using Ca^2+^, Fe^3+^, or using the six phosphate groups of multivalent sodium phytate.^31^^,^^32^^,^^33^^,^^34^^,^^35^ In another study chemical crosslinker free cryosponges have been tested for high stability.^36^ Hybrid bilayer wound dressings have also been prepared to heal infected wounds.^37^^,^^38^ Cyclodextrin and alginate have also been researched for wound healing applications.^39^ Islam et al. have studied crosslinker free collagen for corneal regeneration.^40^ Zhang et al. used crosslinker free bioink for 3D printed hydrogels, which can be used for cartilage regeneration.^41^ All these previous literatures have fabricated hydrogels using ionic crosslinking, host-guest interaction or polymer functionalization. The focus has been to modify the material properties of the hydrogel in a very short time span to deliver the best end results. Due to this, it became difficult to understand the role of adding each ingredient, the changes the polymer undergoes after their addition. We focused on understanding these changes. Das et al. have studied the specific ion effect on hydrogels. The Hoffmeister mediated electrostatic effects have been studied on hydrogel.^18^^,^^42^ They have also formulated a polymeric interaction-based approach to design biomaterials that perform better than marketed products.^43^ A plethora of research has been conducted to design different biomaterial for separate applications. However, the question can be what if there is a possibility to generate a single formulation that can bring different functionalities based on the therapeutic need. This could be a comprehensive approach to design biomaterials and the entire search space of biomaterial applications can be obtained in a single platform. This way we can also understand the multiscale properties of the materials. Several underlying mechanistic possibilities can be unearthed as an obvious outcome from such scientific endeavor.

There are plenty of rooms at the bottom. In this case, suitable choices of FDA-approved polymers, plasticizers, and salts can significantly impact the underlying intermolecular forces. Thus, various properties can emerge from the same polymeric system with slight tweaking of the ingredients to cater to multiple application possibilities. The properties of the hydrogel, like gel strength, swelling behavior, and degradation rate, can be tuned to get customized hydrogel for desired applications. The physical forces are dynamic in nature; thus, achieving the stability of hydrogel is challenging yet crucial. Developing stimuli-responsive hydrogel that can serve multipurpose properties is a complex task that requires careful consideration of the fundamental forces in the polymer domain. A few common intermolecular forces exist, e.g., ionic, dipole, vdW, and H-bonds. By altering these forces, one can impart new properties or features to the hydrogel matrix suitable for a particular application. Researchers are actively exploring a plethora of strategies to fabricate hydrogel using various chemical methodologies and implementing different polymers to prepare hydrogels catering to different properties. We endeavor to relook into this strategy and design hydrogels solely using FDA-approved ingredients without using any chemical crosslinkers. The physical forces within the polymer matrix can be tuned, leading to the development of a wide range of diverse biomaterials using the same polymeric system. Thus, the same formulation can be a generic platform for the design of a variety of biomaterials under the same umbrella. This will reduce the effort of searching for new polymers for different applications and negate the side effects caused by harmful chemicals. The development and approval of biomaterials require compliance with the rigorous regulatory framework of the FDA. This idea ensures the safety, efficacy, and easier clinical translational processes of the hydrogels as we have tried to eliminate the possible toxicity factors such as chemical crosslinkers or surface modifiers, etc. at the design stage. The choice of polymers is of tremendous importance. One polyelectrolyte (PE) (e.g., alginate/chitosan) and one polyampholyte (PA) (e.g., gelatin) with different plasticizers will have a variety of associative possibilities in a suitable formulation medium. These along with common salts (e.g., NaCl) in different proportions, can significantly manipulate the ionic and dipolar forces.^44^^,^^45^ Adding plasticizers like PEG with different molecular weights can lead to direct intervention in vdW and H-bond interactions. Global plasticizers like glycerol have a tremendous capability to interfere with H-bond networks. In short, the choice of materials and their different mole ratios can alter the underlying intermolecular forces and lead to several interesting properties that cater to various applications.

**Figure 1:**
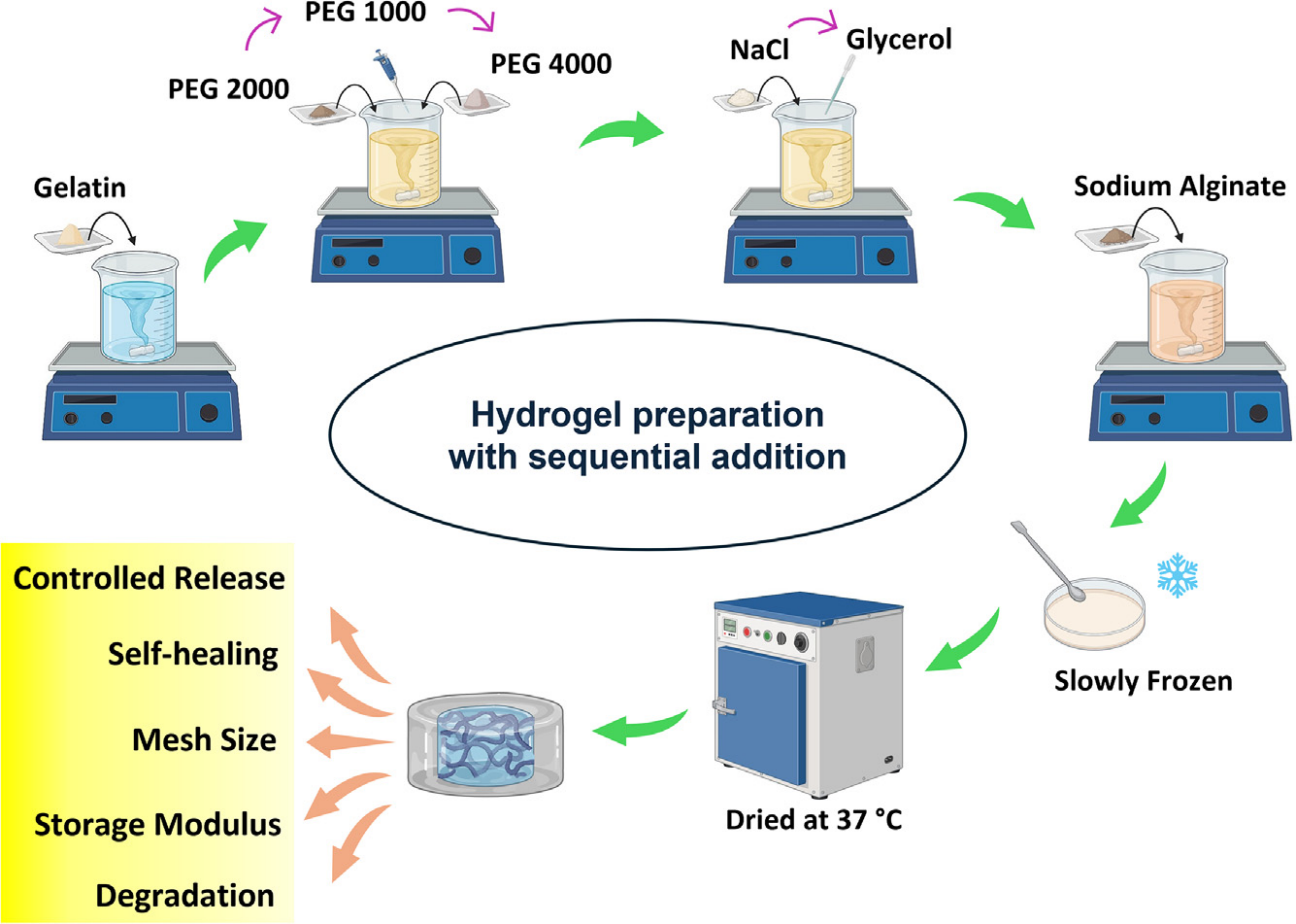
Hydrogel preparation procedure Schematic representation of hydrogel preparation with sequential addition of the ingredients. See also STAR Methods section fabrication of hydrogel.

Instead of searching for new materials/crosslinking methods/functionalization possibilities, the same old FDA-approved polymers, plasticizers and salts can bring the desired changes without any fear of long-term cytotoxicity or regulatory delays. However, stabilizing this physically crosslinked hydrogel is a complex, challenging task and quite an upstream domain. “What to add”, “when to add”, “under what condition to add” and “how much to add”, are the relevant scientific questions in the backdrop of several characterization tools like dynamic light scattering (DLS), FTIR-ATR, UV-spectrometer, rheometer, etc. as well as simulation techniques like molecular dynamics (MD). Such a holistic effort can systematically and scientifically reveal the fundamental features of associated force fields and help in subsequent mapping into the final desirable properties of the polymer formulations. This study brings forward a platform where multiple polymers play a significant role. There is a gap between academic research and industrial practices; translational research can bridge the gap, but multiple barriers are difficult to mitigate. The macro-level properties of these hydrogels, like gel strength, stability, degradation, and self-healing capability, can be tuned by microlevel polymer entanglements using the same ingredients without the need for any chemical crosslinker (Figure 1). This research is trying to establish the understanding of utilizing the already approved biomaterials in their native forms within their permissible limits of usage to customize the same formulation toward a variety of application possibilities.

## Results and discussions

### Role of ingredients

The fabrication of crosslinker-free hydrogel requires thorough knowledge of the fundamental forces present in the polymeric matrix. The macroscopic properties can be tweaked using the microlevel multiscale analysis. Ideally, if a particular sample performs poorly in a single property like zero-order release, it is not recommended for any other application. However, here, we have observed that the sample that shows poor zero-order release has a higher percentage recovery from self-healing. Furthermore, the best sample with 24-h zero-order release also behaves moderately well in other properties. Samples with a higher self-healing recovery show faster degradation within 10 days and a higher mesh size. A higher amount of plasticization gives immense flexibility to the gel, thereby increasing the mesh size, whereas lower plasticization gives rigid gels with a small mesh size. The swelling degree and storage modulus are in direct proportion to each other. Hence, we have observed in each case that the poor samples for a particular objective can be proven as good samples for a different property without the need for any additional chemical interventions releasing any toxic byproducts. Thus, we believe this is a one-of-a-kind study that has explored the benefits of every sample in the total search space of data. The theories of neutral and charged polymers primarily discuss dilute concentration. Hence, the behaviors of multiple polymers (>3) are still unknown to the research community. The methodology used to achieve our target was to control the behavior of polymer chains using physical forces without using any crosslinker. Gelatin (G) is a protein (polyampholyte) containing both positive and negative charges, whereas sodium alginate is an anionic polymer (polyelectrolyte). Thus, they form a rigid and stable hydrogel via electrostatic interactions, hydrogen bonds (H-bonds), van der Waals forces (vdW), and hydrophobicity. Here, different FDA-approved ingredients are chosen that can tune these forces. Different molecular weights (Mw) of PEG can tune the vdW forces and, to some extent H-bonds, and glycerol can significantly tailor the hydrogen bond (H-bond). However, one cannot choose a sufficiently high Mw of PEG. A high Mw (like, PEG 6000) may prevent PEG from diffusing between polymer chains, and it will not exhibit similar plasticizing effects. Further, a much lower Mw of PEG was not chosen as the plasticizer may leach out from the gel due to its smaller Mw and faster diffusion.^29^ PEG if added in higher concentration or higher Mw causes phase separation of the gelatin which forms an inhomogeneous distribution of other ingredients in the polymer matrix.^29^^,^^46^^,^^47^ Smaller Mw of PEG was not utilized as it can enter inside the gelatin. We are using glycerol which has hydrogen bond capacity and can enter the gelatin matrix. We have used two different kinds of plasticizers. One is local plasticizer like PEG, which acts mainly in between two different polymers and another is global plasticizer like glycerol, that penetrates everywhere owing to its smaller size to fine-tune the hydrogen bonds. NaCl will dissociate into Na^+^ and Cl^−^, imparting its electrostatic effect on the structure of the charged polymers. Thus, NaCl can alter the electrostatic forces of the polymer solution. The concentration of NaCl and glycerol was chosen based on our previous experiences. A higher concentration of salt causes fluctuations in gelatin due to unfulfilled hydration layer and may result in distorted hydrogel which is not desirable.^48^^,^^49^ Glycerol if added in higher concentration leaches out sacrificing the stability of the hydrogel.^46^ The system is correlated due to long-range electrostatic forces, and every molecule knows the presence of others. These forces are non-linearly coupled to each other and not yet fully understood. Hence, this work creates an opportunity to understand these forces and their effects on biomaterials.

Rather than attempting to investigate strategies to develop various materials for desired properties, we focused on addressing fundamental questions, such as whether the same formulation could be used to design hydrogel with different properties. We investigated whether the properties of hydrogel can change from one behavior to another in the presence or absence of different plasticizers and salt. Thus, the potential of physically crosslinked SA/G hydrogels was tested in the presence of PEG 2000 (P2K), PEG 1000 (P1K), PEG 4000 (P4K), NaCl (N), and Glycerol (Gly), all of which are FDA-approved and well within the prescribed concentration limits. A full factorial Design of Experiments (DOE) was prepared with varying amounts of plasticizer and salt to get a large domain (Table S1). The 72 sample sets were tested for gel strength and swelling degree. The swelling degree tells us about the hydrogel’s water-holding capacity and the hydrogel’s stability at the equilibrium swelling point. Gel strength talks about the strength of the hydrogel when a certain shear strain is applied. Thus, these two parameters were selected to represent the whole domain of strength and stability of the hydrogels under the same umbrella. The total dataset was normalized with respect to the highest quantity to get the same scale for each property and to bring the dataset into a range between zero and one. Hence, the highest quantity will acquire a value of one and the lowest quantity will have a value near zero. As a result, we were able to determine the specific search domain. A two-dimensional (2D) plot of normalized swelling degree (NSD) vs. normalized gel strength (NGS) was designed. Thus, these two parameters were chosen to obtain the different properties of the hydrogel. A clustering analysis was performed, and from each cluster, random representative points were selected for further study of five properties.

i) controlled drug release,
ii) self-healing,
iii) mesh size,
iv) storage modulus, and
v) degradation rate.

**Figure 2:**
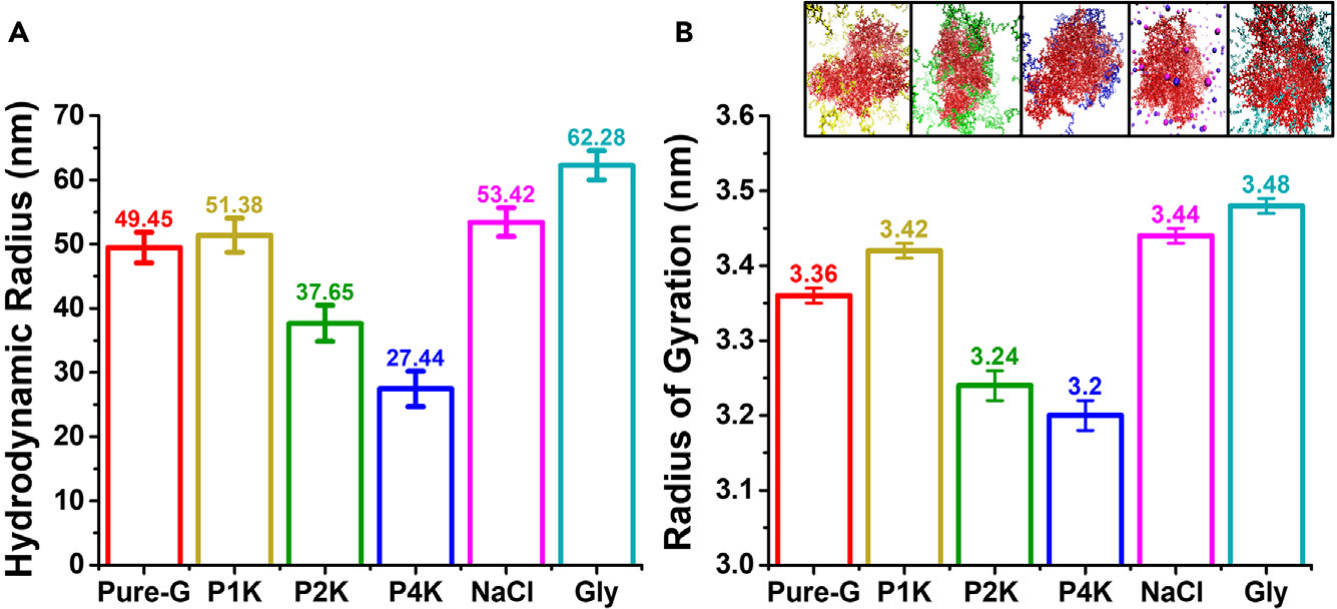
Size of the gelatin polymer P1K (PEG 1000), P2K (PEG 2000), P4K (PEG 4000), Gly (Glycerol). (A) Hydrodynamic radius with the addition of each individual ingredient. Data are represented as mean ± SD of *n* = 3 samples. (B) Radius of gyration with the addition of each individual ingredient. Data are represented as mean ± SD of *n* = 3 samples.

The prime motive is to obtain a variety of biomaterial properties under the same umbrella using the same polymers. Thus, an improved understanding of polymeric interaction is required to achieve programmable performances of the hydrogels. In the previous research, we limited our discussion to the role of plasticizers (PEG 2000 and glycerol) and salt (NaCl) in designing crosslinker-free hydrogel solely for controlled-release application.^46^ However, in the present work, we attempted to establish a generic platform for various possibilities in terms of properties with a broad range of applications. Here, we discuss the release properties as well as other properties like self-healing, mesh size of the hydrogel arising just by changing the ingredients, which has a high potential for diverse applications. To meet this requirement, the role of each ingredient was first analyzed using DLS and molecular dynamic (MD) simulations. Both the DLS experiments, and MD simulations were conducted on gelatin solution to understand the role of the plasticizers and salt on gelatin. In sequential additions, the sodium alginate is added at the last (as a shell); the core polymer matrix is thus having all other ingredients and can determine the major functionality of the biomaterials as evident in the performance studies (e.g. drug release). DLS gives us the diffusivity of gelatin; from these data, the hydrodynamic radius (R_h_) of the gelatin chains can be obtained. This will provide us with knowledge of how gelatin interacts with other ingredients. To validate the trend of R_h_ obtained experimentally and to understand the molecular level interaction MD simulations were performed which gives us the value of radius of gyration (R_g_) of the gelatin. It has been proved that P2K (PEG 2000) acts as a local plasticizer, forming a layer between gelatin and alginate. In contrast, glycerol acts as a global plasticizer as it can diffuse inside the polymer matrix due to its smaller size and higher H-bonding capacity. Here, it is observed that P1K, due to its lower molecular weight compared to P2K, can go inside the gelatin; however, not as much as compared to glycerol. On the other hand, P4K with a heavy molecular weight chain cannot go inside a gelatin matrix like P2K (Figure 2). P4K also promotes phase separation at a very low concentration. Phase separation is quite common in a protein-salt system.^49^ However, phase separation of gelatin has been observed with the addition of plasticizers, which is crucial to studying various disease models.^50^ The addition of NaCl causes a collapse-re-expansion phenomenon.^48^^,^^51^^,^^52^ Initially, when gelatin is dissolved in DI water (pH 6), it contains a few positive charges as the iso-electric point (IEP) for gelatin being used is around 8. Thus, the salt ions first screen those positive charges, collapsing the chain. Further addition of excess salt leads to expansion of the chain as the electrostatic attraction within the chains gets screened. However, here, the salt concentrations used are higher than the collapse-re-expansion point; hence, only an expansion is observed.^51^ Thus, from the previous findings, it can be justified that.

(i) P2K, P1K, and P4K promote mainly the local plasticization,
(ii) the addition of P4K causes phase separation of gelatin chains,
(iii) NaCl yields expansion of the chains, and
(iv) glycerol acts as a global plasticizer.

**Figure 3:**
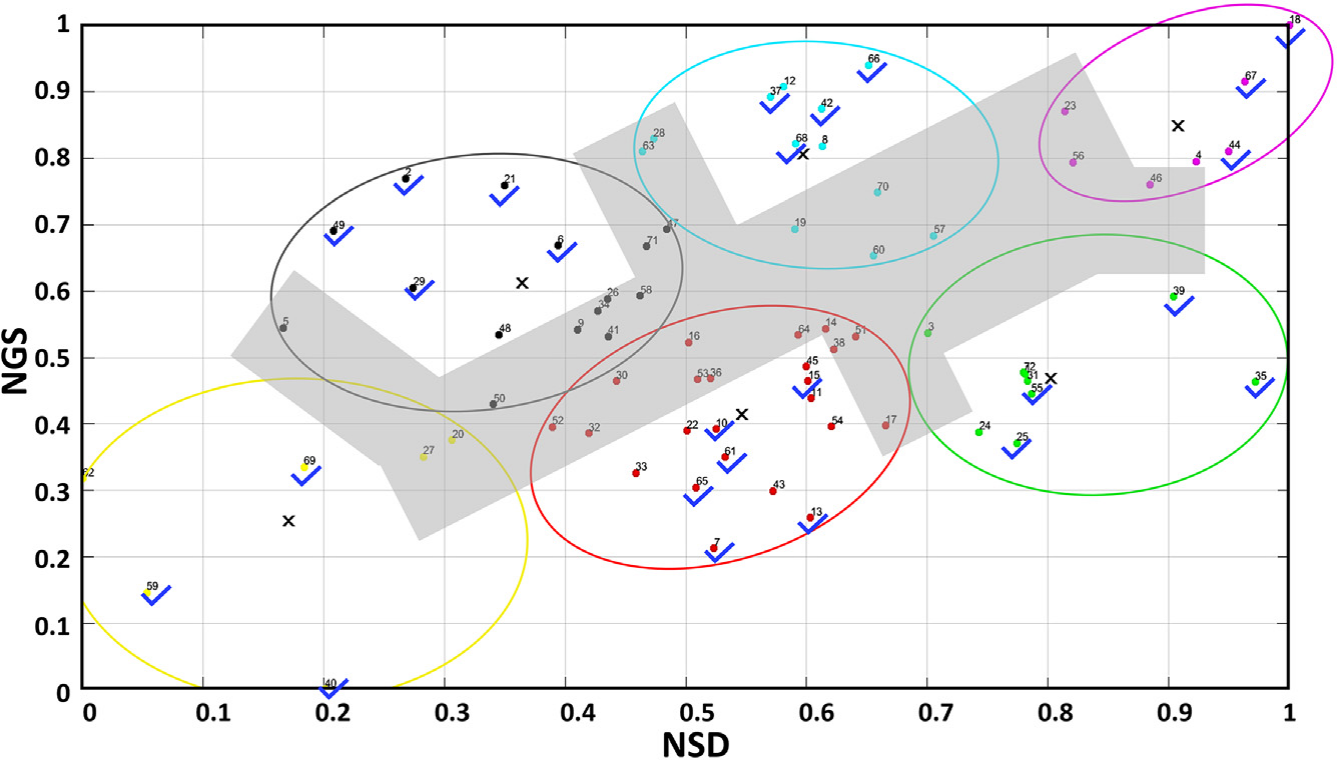
Two-dimensional (2D) plot of NSD vs. NGS The colored circles depict the six clusters and the blue tick-marks are the representative points. The results are obtained from *n* = 3 replicates and normalized with respect to the highest value. The data are statistically significant (*p* value <0.05). See also Tables S1 and S2.

The role of different ingredients depicted in Figure 2 helped us to ask the next question. The next question was to find the possibility of different properties within the total datasets. As mentioned earlier, the swelling degree and gel strength analysis was performed for 72 sets of samples. Next, from these 6 clusters, 25 representative points were tested for different properties. To exclude the interference between the clusters, the boundary points of the clusters were not considered, which was done manually (Figure 3; Table S2). The five different properties represented are.

i) controlled drug release,
ii) self-healing,
iii) mesh size,
iv) storage modulus, and
v) degradation rate.

These different properties can dictate the preliminary requirements of the hydrogels for different applications like drug delivery, tissue engineering, wound healing, bone repair, etc. In the next sections, we will discuss these properties, respectively.

### Controlled release

The possibility of a long hour *in vitro* zero-order release was tested for oral drug delivery application. A model drug, naproxen sodium, was used, which is an NSAID. A single parameter was used to analyze the drug release, including the amount of drug released until zero-order was observed and the time taken for zero-order release (Figure S1). Thus, the drug release data were represented using a single parameter: release fraction (RF) as described earlier (Equation 2).^44^^,^^46^ These RF data were normalized with the highest value. Each property was normalized to obtain a similar scale of 0–1.

**Figure 4:**
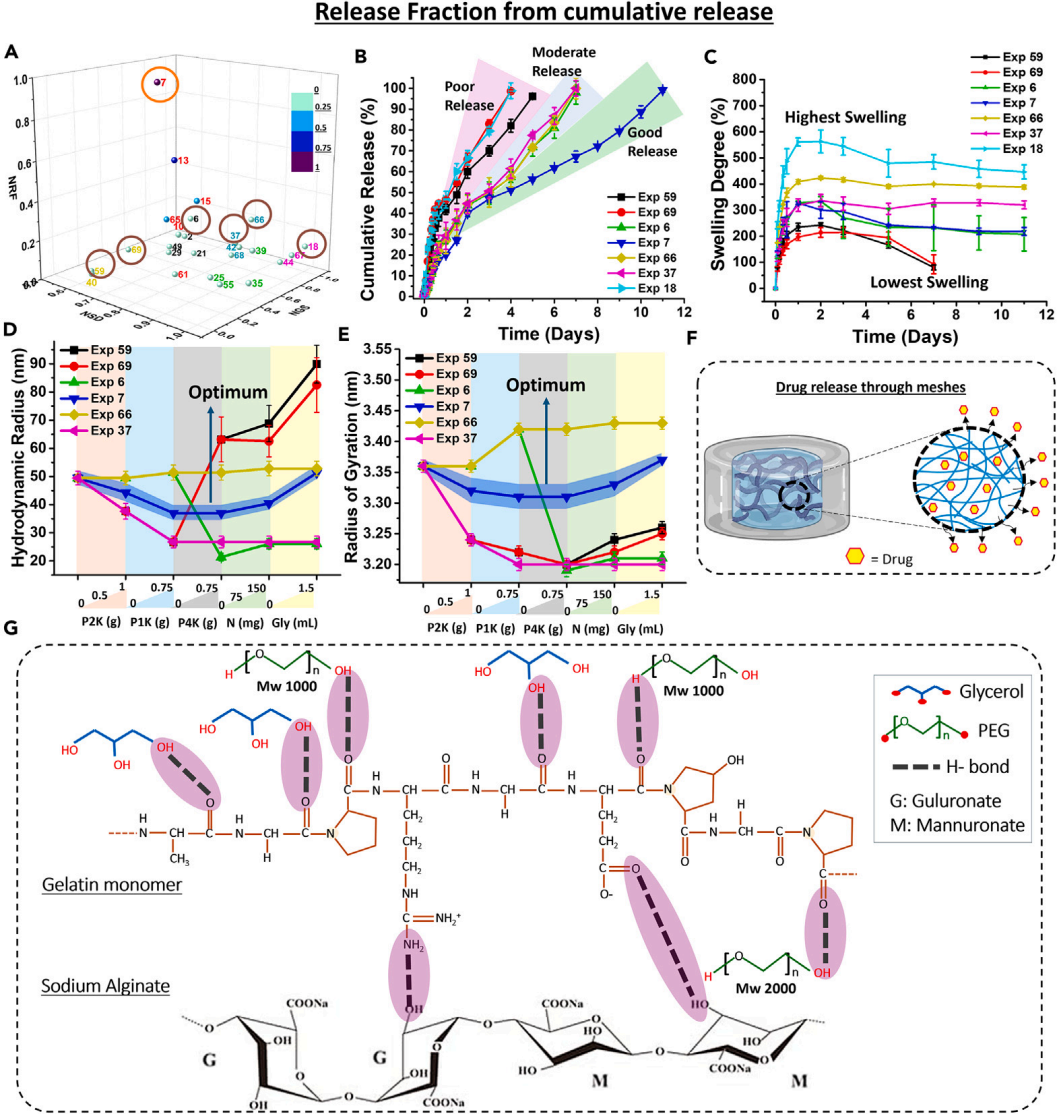
Release fraction from cumulative release (A) Three-dimensional (3D) plot of NRF, NSD and NGS; (B) Cumulative release profiles; (C) Swelling degree curves; (D) Hydrodynamic radius; (E) Radius of Gyration; (F) Schematic representation of drug release; (G) Hydrogen bond mechanism for Exp 7. Data are represented as mean ± SD of *n* = 3 samples (B–E). The data are statistically significant (*p* value <0.05). See also Figure S1.

The normalized release fraction (NRF) shows the highest for, the sample consisting of PEG 2000, PEG 1000, NaCl, and Glycerol (Exp 7). A 3-D plot of NRF, NSD, and NGS was plotted to understand the relation between these properties. The sample lies in the middle range in all the properties and has moderate values of NSD, NGS, percentage recovery, storage modulus, mesh size, and low degradation. Thus, it follows an optimum path for each of these properties, including gel strength and swelling degree (Figure 4A). Thus, neither too high NSD or NGS nor too low NSD or NGS can provide a good NRF. Thus, this sample (Exp 7) was named the optimum drug delivery sample. Higher NSD or NGS was observed for samples with low plasticization. However, a higher amount of plasticization leads to a low value of NSD or NGS, causing a poor release (Figure 4B). To explain this phenomenon, a few points are chosen (one optimal and the rest sub-optimal).

Table 1 shows the swelling degree obtained for various other hydrogel formulations. It can be inferred from this table that biopolymers like gelatin, alginate are widely used for different applications like drug delivery, tissue engineering, wound healing etc. They exhibit a wide range of swelling degrees starting from 6% to 2100% in some chemically modified polymers. Therefore, when compared with the literature it can be said that the swelling degree of prepared hydrogels are comparable to physically crosslinked hydrogels. Here, without using any chemically modified polymers we could show a varied equilibrium swelling degree of polymers ranging from 215% to 484% (Figure 4C).

**Table 1:** Swelling degree obtained in various other hydrogels

Hydrogel Formulation	Swelling Degree	Literature
Soy Protein Isolate, Collagen & Sodium Alginate	100–130% (different compositions)	Esmaeili et al.^37^
Copovidone & Polyvinyl Alcohol	183–981% (different compositions)	Kim et al.^38^
(2-Hydroxy isopropyl)-β-cyclodextrin (HPβCD) & Alginate	200–450% (different compositions)	Mohammadi et al.^39^
Sodium Alginate & Gelatin	50–250% (different compositions)	Bhutani et al.^29^
Polyacrylamide-m-g-alginate co-polymer & carboxymethyl cellulose	400–600% (different compositions)	Kulkarni et al.^53^
Sodium Alginate & Polyvinyl Alcohol	50–650% (different compositions)	Hua et al.^54^
Alginate-polyacrylamide graft copolymer	100–200% (different compositions)	Tripathi et al.^55^
Alginate & Carboxymethyl guar gum	200%	Bajpai et al.^56^
Alginate & Gellan gum	6–15% (different compositions)	Jana et al.^57^
Gelatin & Sodium Alginate	200–300% (different compositions)	Das et al.^42^
Gelatin & Chitosan	400–1200% (different compositions)	Koc et al.^58^
Chitosan wound dressing	300–2100% (different compositions)	Kong et al.^59^
Gelatin Methacryloyl & Sodium Alginate	75–125% (different compositions)	Ma et al.^60^

The hydrogel preparation procedure includes the addition of all the components in a gelatin liquid solution, followed by the drug naproxen sodium and sodium alginate, which leads to a rapid gel formation. Thus, all the components have enough time to properly interact with gelatin forming the core of the hydrogel and finally, sodium alginate acting as a shell on top of it. Hence, a DLS experiment was performed following our previous protocol, where a sequential analysis was done after the step-by-step addition of each ingredient (Figure 1), and the R_h_ was calculated from the diffusivity values of the polymer. The reason for adopting sequential addition characterization was 2-fold. Firstly, the polymer, here gelatin attains a stable state only after 30–45 min of addition of plasticizers or salt.^49^^,^^61^ Secondly, the sequential characterization was done to exactly mimic the hydrogel fabrication protocol. This technique surfaced the in-depth polymeric interaction during the hydrogel preparation.^18^^,^^43^^,^^46^ Here, a restructuring of the gelatin chains was obtained, and the final structure was able to regain its initial structure after passing through a collapse and re-expansion (Figure 4D). Figure 4B shows the drug release of a few selected experiments. The findings suggest that experiments with a high number of ingredients (Exp 59, 69) or those made entirely of SA/G hydrogel (Exp 18) show a very fast release with the entire drug released within 5 days. Hence, both extremes perform poorly, and this is due to improper drug distribution as the polymer chains are too open (Figure 4D). On the other hand, experiments with the decreased value of R_h_ (Exp 37, 6) show a moderate release profile. Furthermore, even if the final R_h_ value localizes around the initial R_h_ value (Exp 66) which is similar to the final value of optimum sample (Exp 7), an inadequate release profile is obtained. This could be due to the collapse and re-expansion phenomenon of the R_h_, which can be only observed for the optimum sample (Exp 7). The trend of R_h_ obtained experimentally was also validated using MD simulation. The MD simulation has also shown a similar collapse-re-expansion phenomenon, where the final polymer size regains the initial value (Figure 4E). Thus, the condition for obtaining a better release profile could be justified as a moderate value of hydrogel properties like swelling, gel strength, storage modulus, self-healing, mesh size, degradation, and the collapse-re-expansion of the polymer chains restructuring them to a final value closed to the initial one. Figure 4F shows a schematic of the drug release through the polymer matrix. The possible mechanism of H-bonding between the polymers is depicted in Figure 4G. To the best of our knowledge, this kind of holistic revelation and approach of polymer restructuring is unparalleled in the entire drug delivery literature.

### Self-healing properties

**Figure 5:**
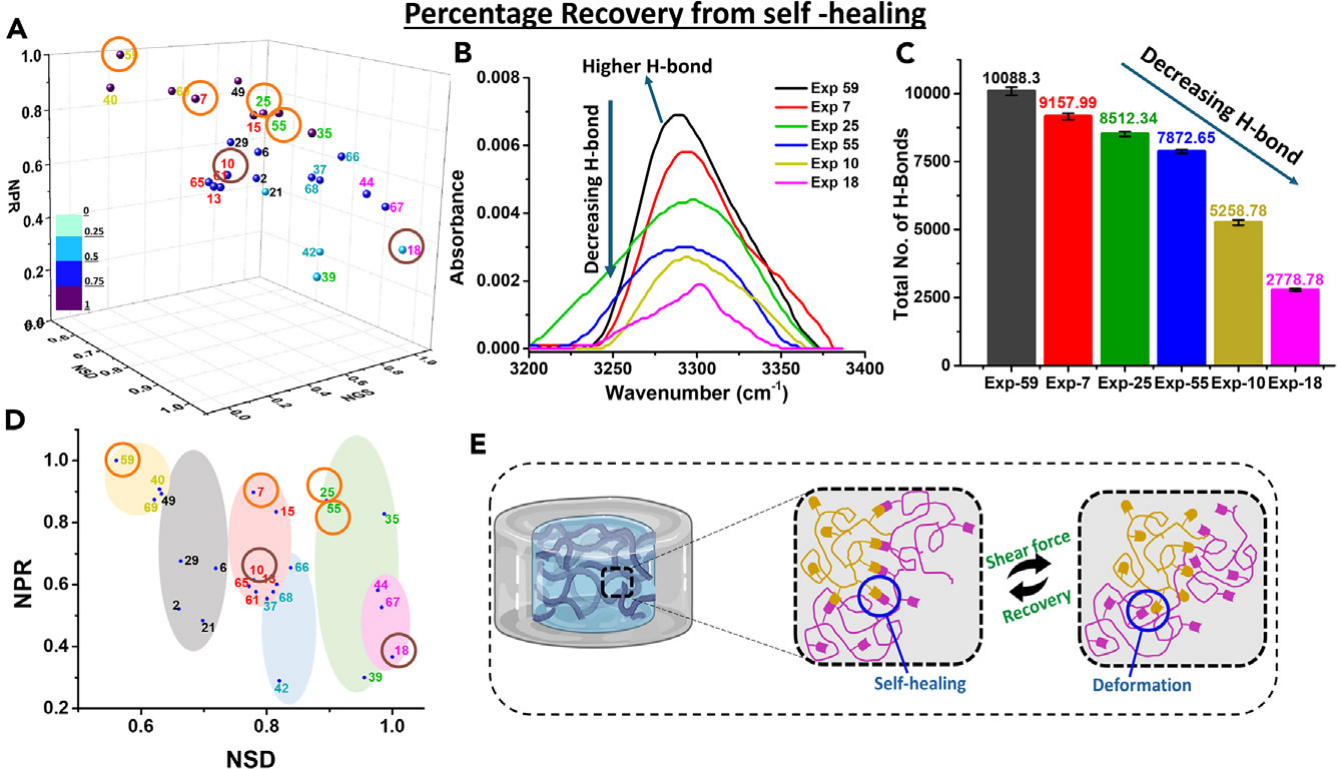
Percentage recovery from self-healing (A) 3D plot of NPR, NSD, and NGS; (B) FTIR-ATR spectra for 3000-4000 cm^−1^; (C) Total number of H-bonds obtained from MD simulation. Data are represented as mean ± SD of *n* = 3 samples; (D) 2D plot of NPR vs. NSD showing the same clustered points obtained previously; (E) Schematic representation of self-healing. The data are statistically significant (*p* value <0.05). See also Figure S2A.

The alternate step strain measurement curves of the swelled hydrogels were analyzed after 24 h to show their self-healing behavior. Figure S2A shows the alternate step strain measurement curves. Hydrogels break their structure at higher strain and regain their structure after removing the high strain, which shows the self-healing properties of the gel. The percentage recovery of the gel structure after breaking its structure from the initial state is calculated and normalized with respect to the highest value, giving normalized percentage recovery (NPR). A 3D plot was generated using NSD (swelling degree), NGS (gel strength), and NPR (percentage recovery) to represent the important properties of the hydrogel and to choose the best among them. Self-healing properties of hydrogel can be modulated by multiple factors like electrostatic interactions, H-bonding, hydrophobic interactions, metal-ligand complex, protein self-assembly, etc.^42^^,^^62^ However, the major contributing factor here could be the H-bonding as different plasticizers have been used. It can be inferred from Figure 5A that, samples containing a higher amount of plasticizer (Exp 59) have a higher recovery percentage of 63% (Table S3).

To further understand this, the FTIR-ATR analysis was performed on a few samples with varying levels of recovery (Figure 5B). The broad absorption band in the range of 3000–4000 cm^−1^ represents the coupled N-H and O-H stretching vibration. Figure 5B shows that Exp 59 has the highest absorption thus confirming higher H-bond formation. This suggests that H-bonding is the dominating factor here, for higher percentage recovery. This is because of the roles played by multiple plasticizers altering the H-bond network of the hydrogel. Thus, a higher percentage recovery can be observed for samples with higher H-bonds. As the percentage recovery decreases, the intensity of the 3000–4000 cm^−1^ also decreases for other samples (Exp 59 > Exp 7 > Exp 25 > Exp 55 > Exp 10 > Exp 18). Moreover, the total number of H-bonds between gelatin, plasticizer, and water as obtained from MD simulation, also follows a similar trend (Figure 5C). Thus, a hydrogel with good H-bonding capability exhibits better percentage recovery after applying higher strain. The samples with poor NRF values (Exp 59 and 40) have higher percentage recovery (Figure 5D). Moreover, the optimum sample obtained from NRF (Exp 7) shows a good recovery, proving that a good self-healing capability can provide better drug release. This kind of self-healing hydrogel has an attractive application for wound dressing, bone tissue engineering.^63^^,^^64^ We want to emphasize that one need not explore other methods, such as surface modifications or chemical crosslinking, to achieve various properties for different materials. The same polymeric network can provide numerous opportunities for material diversity as long as the intermolecular forces can be tuned well within a simple framework.

### Mesh size

**Figure 6:**
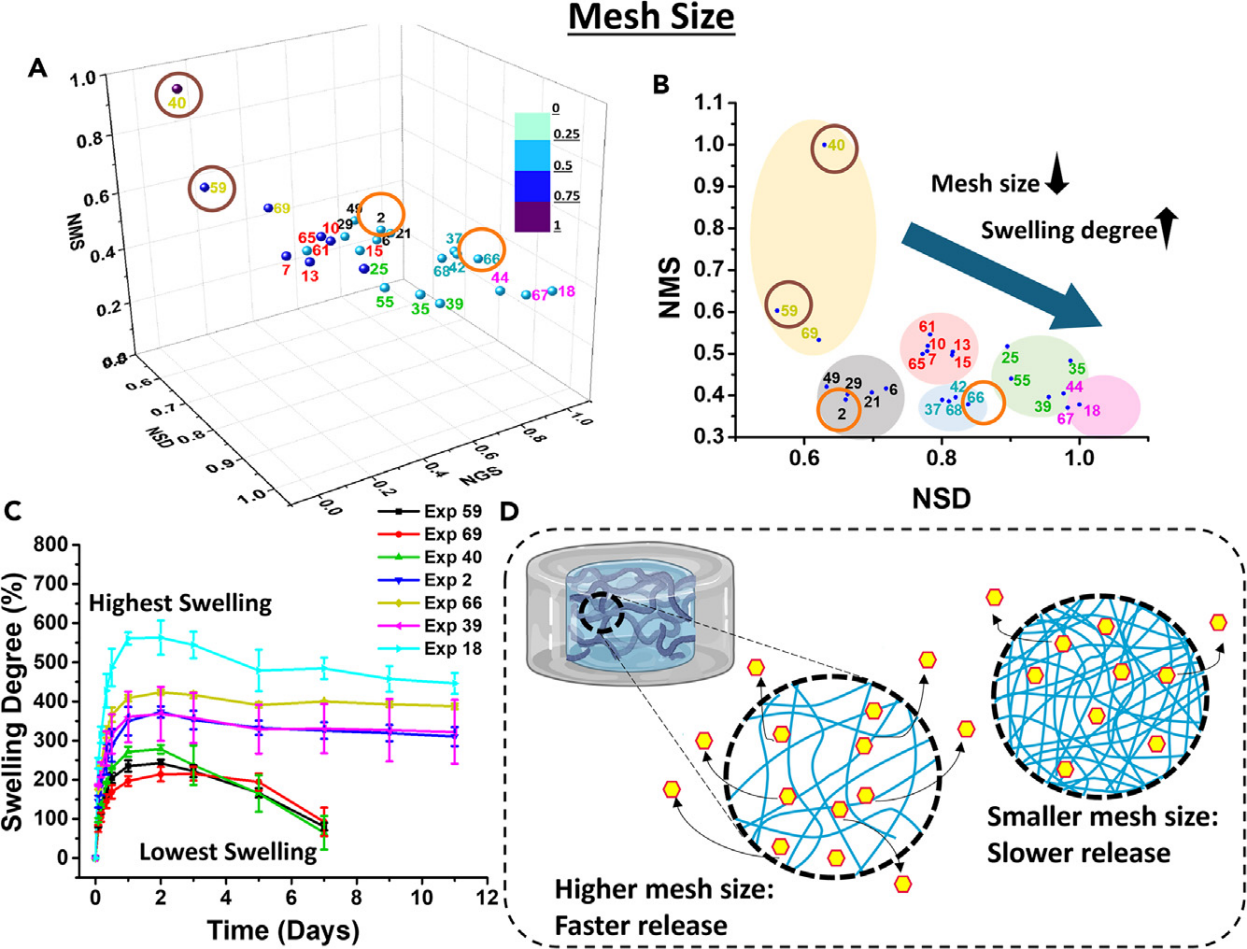
Mesh size of the hydrogels (A) 3D plot of NMS, NSD, and NGS; (B) 2D plot of NMS vs. NSD showing the same clustered points obtained initially; (C) Swelling degree curves. Data are represented as mean ± SD of *n* = 3 samples; (D) Schematic representation of mesh size and its impact on drug release. The data are statistically significant (*p* value <0.05). See also Table S3.

The rheological analysis of the hydrogels was conducted to determine the mesh size and storage modulus of the hydrogel and check their suitability and possible application for tissue engineering, scaffolds, wound healing, etc. The hydrogels were subjected to an amplitude sweep test, and the linear viscoelastic region (LVER) was noted. A frequency sweep analysis was performed using 0.1% strain within the linear viscoelastic region. Figure S2B and S2C show the results obtained from amplitude sweep and frequency sweep, respectively. It can be observed that storage modulus is higher than the loss modulus depicting the gel-like structure. The mesh size was calculated and normalized with respect to the higher value. Samples with a higher plasticizer content show a higher normalized mesh size (NMS) (Figure 6A). This is because gelatin is more plasticized in this case; thus, gelatin is not able to interact with alginate due to multiple layers of plasticization. The crosslinked network has more open spaces and a larger mesh size. Figure 6B portrays that the trend observed for the mesh size is opposite to that of the swelling trend. This essentially means that samples with larger mesh size have lower swelling degree, which inhibits the diffusion of media (PBS) inside the hydrogels. The samples with higher mesh sizes (Exp 59 and 40) also have higher percentage recovery. Due to the same reason, the mesh size exhibited a decreasing trend with increasing swelling degree. The swelling degree will be higher when plasticization is less, and mesh size will be low (for example Exp 18, Exp 44, and Exp 67). The highest swelling degree is observed for the pure hydrogel (Exp 18) and lowest is observed for the sample with the highest amount of plasticization (Exp 59) (Figure 6C). When the mesh size and swelling degree are plotted for the representative points, the same clustered points exhibit their earlier relation. Larger mesh size also gives a burst release as the drug molecule can easily diffuse through it (Figure 6D). For example, Exp 40 and 59 show larger mesh sizes and perform poorly in drug delivery. Samples with less plasticizer show a lower mesh size as there is more crosslinking between gelatin and alginate chains. Here, the chains are densely packed, leaving fewer open spaces. However, the drug release profile is unsuitable for these samples as they have a very high swelling capacity; thus, excess PBS is entrapped within them, and thus, higher drug dissolution can occur with time. Therefore, the optimum sample of NRF (Exp 7) has a moderate mesh size value, providing a good release profile.

The hydrogels are prepared without using chemical crosslinkers, which can lead to the release of toxic byproducts over time. Furthermore, their biodegradable nature and variety of mesh sizes can have potential applications for wound healing, which can permit the transport of bioactive molecules.^65^ However, separate research and detailed analysis need to be done for the same. A single-factor ANOVA analysis of the data with 95% confidence, revealed that different plasticizers and salt have an impact on the equilibrium swelling degree, gel strength, drug release, percentage recovery, mesh size, storage modulus, and degradation of the hydrogels (Table S4).

### Storage modulus

Hydrogels were investigated using amplitude sweep experiments to find their linear viscoelastic region (LVER). The storage modulus was observed to be higher than the loss modulus in this region, indicating that hydrogels remained in their elastic (solid-like) region (Figure S2B).

**Figure 7:**
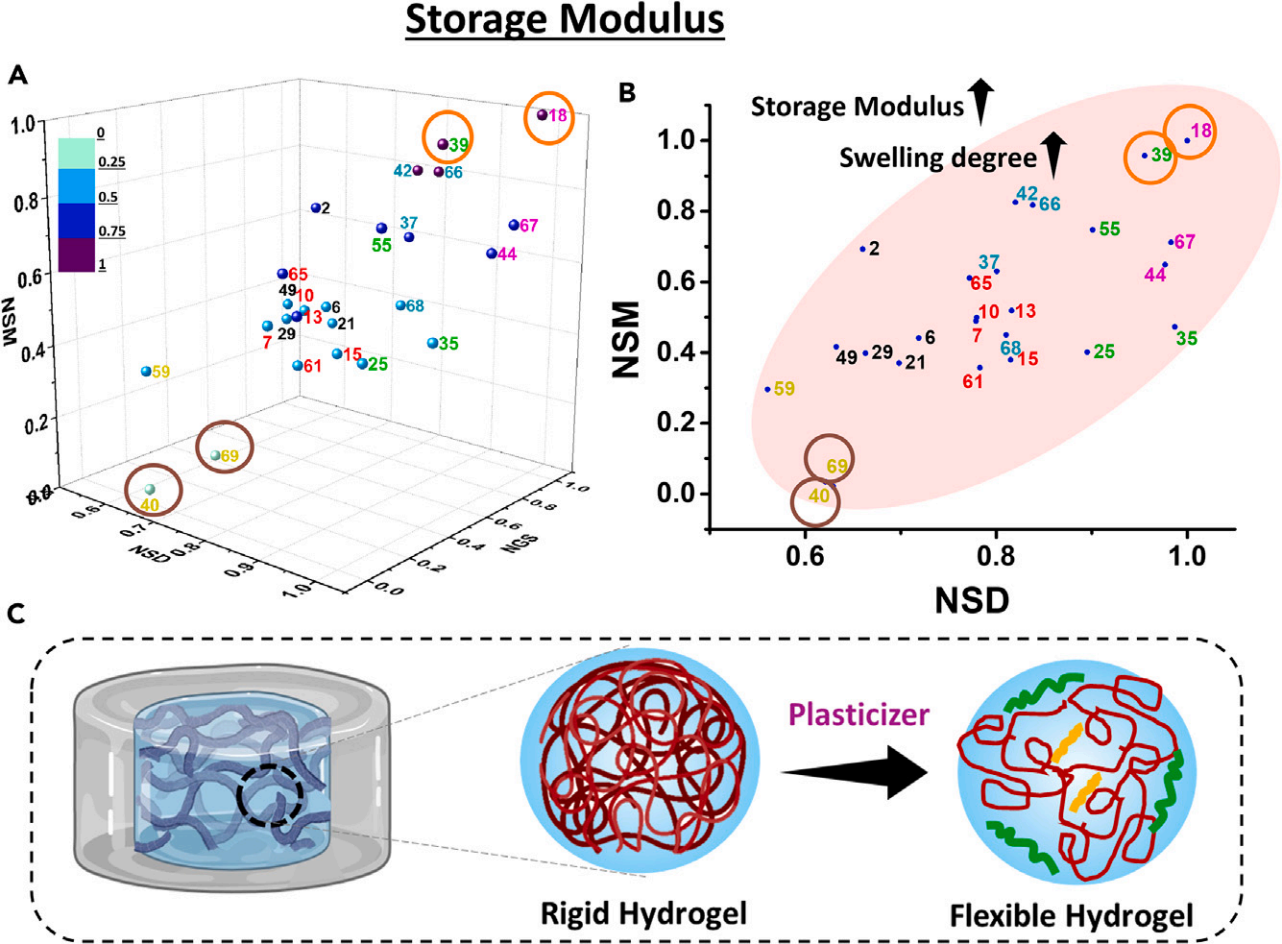
Storage modulus of the hydrogels (A) 3D plot of NSM, NSD, and NGS; (B) 2D plot of NSM vs. NSD; (C) schematic representation of rigid hydrogel and flexible hydrogel with the addition of plasticizers. The data are statistically significant (*p* value <0.05). See also Table S3 and Figures S2B and S2C.

From Figures S2B and S2C it can be inferred that samples with less plasticizer show a higher storage modulus (33 kPa) (Table S3). Therefore, due to the stronger interactions between gelatin and alginate in the case of less plasticized samples, the hydrogels form a compact structure that enhances the storage modulus. The storage modulus decreases with increasing plasticizer components (Figure 7A). The storage modulus also exhibited direct proportionality to the swelling degree (Figure 7B).

The samples with less plasticizer have exhibited lower mesh sizes. These samples are also much more rigid and thus have a non-homogeneous drug distribution. Thus, samples with higher plasticizer content have low storage modulus, are flexible, and have a more homogeneous drug distribution (Figure 7C). When the normalized storage modulus (NSM) was plotted against NSD, the optimum sample obtained from NRF (Exp 7) was observed to present in the middle range (∼0.5 NSM). The samples with poor NRF (Exp 59, 69) show a flexible nature with low storage modulus. Samples with higher storage modulus (Exp 18, 39) have low NRF, percentage recovery, and mesh size. Along with the direct correlation of storage modulus with controlled release, these hydrogels have the ability to be suitable for use as a scaffold, bone tissue repairing.^63^^,^^66^^,^^67^ Thus, the experiments revealed that using the same ingredients, we can obtain a diverse range of samples with varying properties, opening up a world of possibilities for scientists and researchers in various fields.

### Degradation rate

**Figure 8:**
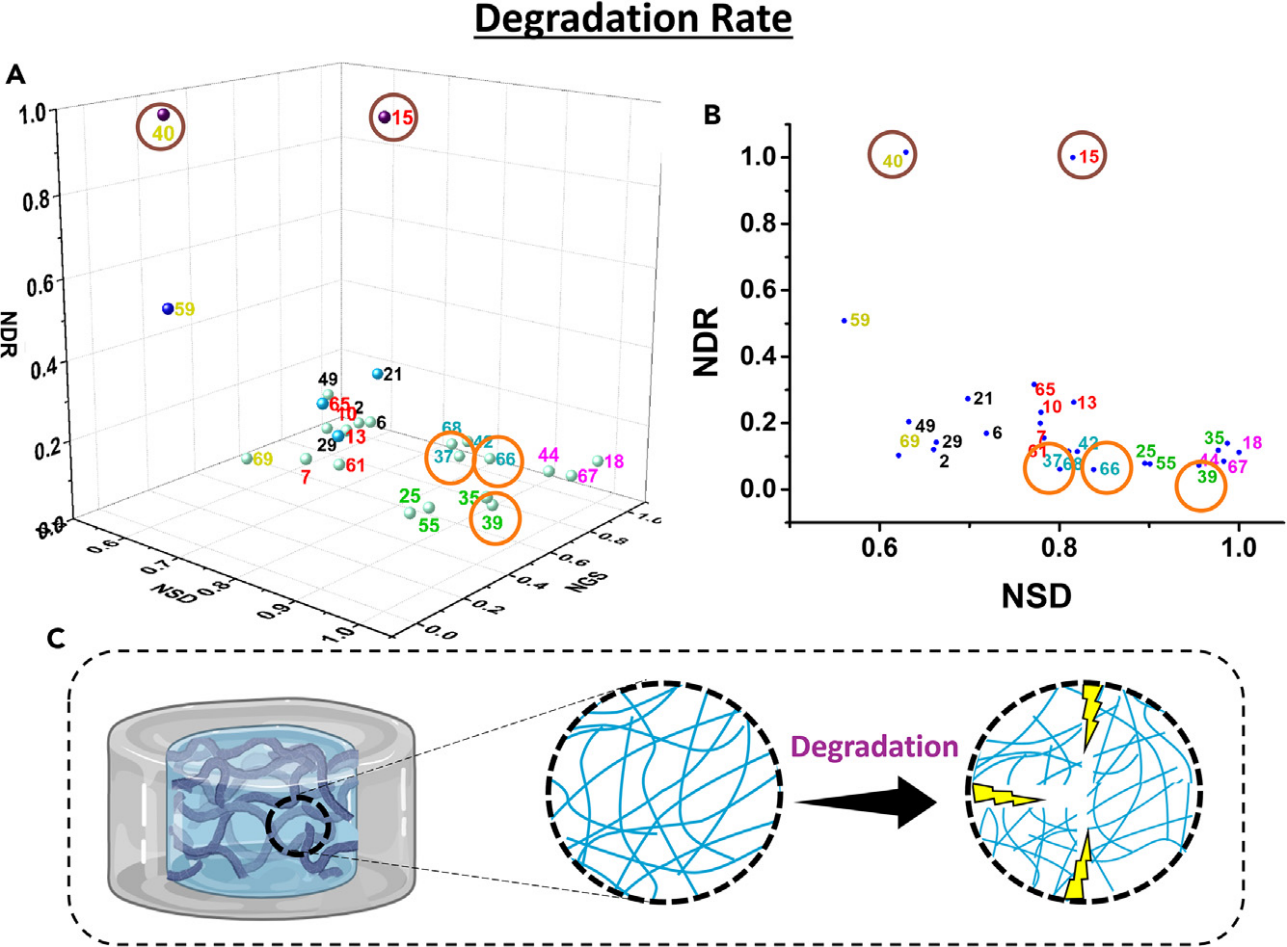
Degradation rate of the hydrogels (A) 3D plot of NDR, NSD, and NGS; (B) 2D plot of NDR vs. NSD; (C) schematic representation of degradation of hydrogel. The data are statistically significant (*p* value <0.05). See also Figure S3 and Table S4.

The stabilities of the hydrogels were also investigated using degradation studies of the gel. The hydrogel samples were immersed in phosphate buffer saline (PBS—pH 7.4), and the weights of the hydrogel were measured. The hydrogels reach their equilibrium swelling degree after 2 days. The degradation percentage of the samples was calculated. The hydrogels (Exp 37, 66) showed only 11% degradation after 19 days (Figure S3A). The hydrogels were intact and degraded gradually, which can be beneficial and is a required trait for application in tissue engineering. As the amount of plasticizer and salt increases, the degradation rate increases, showing a faster degradation and dissolution of the matrix. The degradation percentage for all the samples varies from 11% (good performance), gradually increasing to 80% and 100% for Exp 40, 15 (poor performance) (Figure S3). Similarly, samples with lower degradation percentages (Exp 37 and 66) show lower degradation rates. The samples with a higher amount of plasticization show a faster degradation rate within 10 days (Figures 8A and 8B). However, samples with good performance in the degradation study show poor performance in the controlled release study proving the earlier fact that samples behaving poorly in a specific study can depict an excellent performance in other properties.

These encouraging results clearly illustrate that there is a scope to unearth the potential of native biopolymers without any chemical modifications to deliver a range of applications from a single formulation. Thus, it can be inferred that a variety of samples with different properties can be obtained with the same ingredients just by tweaking the fundamental forces in the polymer domain rather than searching for new materials. In each of these cases, it has been observed that the samples with poor performance in one property have relatively higher performance in another property justifying the central theme of this research. For instance, we have sample with 24 h controlled release and an extended release till 10 days (Exp 7), another hydrogel has a 63% of recovery from self-healing analysis (Exp 59). Additionally, we observed a high storage modulus of 33 kPa in Exp 18 and 39. We also developed a hydrogel that showed only 11% degradation in 19 days (Exp 37, 66). Furthermore, different mesh size options are also available for various possible applications. Different applications demand different features and prioritizing one may come at the expense of others. It is evident that an optimal collapse and re-expansion of polymer chains are imperative for effective drug encapsulation and controlled release. Furthermore, a higher number of hydrogen bonds are essential for a superior percentage recovery in terms of the self-healing properties of the hydrogel. This implies that a sample’s self-healing capability is enhanced at the cost of its controlled release ability. Additionally, a larger mesh size of the hydrogel will result in faster drug release, while a very small mesh size will lead to inadequate drug delivery, both of which are undesirable. Thus, an optimal mesh size between these two extremes is crucial for controlled drug delivery. This indirectly underscores the significance of the precise amount of components required to fine-tune the forces present in the hydrogel. Hydrogels devoid of any plasticizer will yield a rigid hydrogel with the highest swelling capacity, while an increased amount and number of plasticizers can render the hydrogel flexible. This is due to the breakage of hydrogen bonds between the polymers and the formation of new hydrogen bonds between the polymers and the plasticizer, providing the necessary pliability to the system. Hence, a sample with the highest degree of swelling cannot be a flexible hydrogel. This theme forms the heartwood of this research.

### Conclusion

In conclusion, this study suggests that different hydrogel properties can be obtained using the same polymer matrix by slightly tweaking the ingredients. The primary focus was to develop physically crosslinked hydrogel using FDA-approved ingredients for various applications. Instead of inducing new functionalities in the polymers, the physical forces of the polymers can be tuned using different molecular weights of PEGs, salt, and glycerol to get the desired product. Here, five different properties of the hydrogels were evaluated: controlled release, self-healing, mesh size, storage modulus, and degradation rate. It can be inferred that an optimum polymer restructuring helps in a controlled release. The sample with 24 h controlled release and extended release till 10 days (Exp 7) shows a moderate value for gel strength, selling degree, self-healing, mesh size, storage modulus, and degradation rate. Samples showing poor release profiles (Exp 59) are better in terms of self-healing with 63% recovery and flexibility. This sample also has the highest hydrogen bonding capability. Samples with a higher percentage recovery show higher mesh size and an early degradation. Samples with higher storage modulus (33 kPa, Exp 18 and 39) are rigid enough to show a lower mesh size. Only 11% degradation was observed in 19 days for Exp 37 and 66. The complex physical forces associated with the polymeric matrix are all nonlinearly coupled; thus it makes difficult to theoretically calculate these forces in presence of multiple components. The dominating force can be identified in future study for each application separately using different set of experiments. It’s a well-known realization of the field that only negligible research propositions are finally being medically approved for human usage. Use of chemical crosslinkers/functionalization will not only increase the potential risk of long-term toxicity, but also will go through a long regulatory procedure causing a significant delay. Thus, it is prudent to strategize the research efforts within the regulatory approved limits without compromising the functional requirements for an application. Intense studies of molecular interactions with well-proven biomaterials within their permissible limits can unearth many possibilities that are unknown to us so far. This research not only bridges this gap but also shows the tremendous capacity of charged polymers for a variety of possible applications in biomaterials.

### Limitations of the study

Despite the good performance of the hydrogels, there are still some limitations. Although significant effort has been made to illustrate a wide variation of properties using similar polymeric matrices and formulations, their translation into an *in vivo* model is essential. The hydrogel is enriched with multiscale nonlinearly coupled physical forces. This can be extended to study the influence of each force. Each property can be potentially enhanced in separate studies to understand the role of dominating forces. In the current study, the use of hydrogel is limited to demonstrating multiple properties; in the future, they can be studied further for deeper wound healing and tissue regeneration.

## Resource availability

### Lead contact

Further information and requests for resources and reagents should be directed to and will be fulfilled by the lead contact, Saptarshi Majumdar (saptarshi@che.iith.ac.in).

### Materials availability

This study did not generate new unique reagents.

### Data and code availability

• All data reported in this paper will be shared by the lead contact upon request.
• This paper does not report original code.
• Any additional information required to reanalyze the data reported in this paper is available from the lead contact upon request.

## Acknowledgments

The authors would like to acknowledge IIT Hyderabad for providing research facilities. T.B. would like to acknowledge Ministry of Human Resource and Development (MHRD). The authors acknowledge the financial approval from DST (Dept. of Science & Technology, Govt. of India) funding CRG/2021/001039. We also thank the High-Performance Computing Center (HPC) Department of Chemical Engineering of the Indian Institute of Technology, Hyderabad, for providing the computational resources. The authors would like to acknowledge BioRender.com. Part of the schematics was prepared with the help of BioRender.com.

## Author contributions

T.B.: conceptualization, formal analysis, methodology, writing—original draft, software, data curation, validation, visualization, investigation. D.G.: formal analysis, validation, visualization, investigation. S.M.: conceptualization, supervision, writing—review and editing, funding acquisition, resources, project administration. The manuscript was written through contributions of all authors. All authors have given approval to the final version of the manuscript.

## Declaration of interests

The authors declare no competing interests.

## STAR★Methods

### Key resources table

**Table:** 

REAGENT or RESOURCE	SOURCE	IDENTIFIER
**Chemicals, peptides, and recombinant proteins**		

Gelatin	Sigma Aldrich	cat#G2625; CAS: 9000-70-8
Sodium alginate	ThermoFisher Scientific	cat#ALF-J61887-22, CAS: 9005-38-3
Polyethylene glycol 2000	ThermoFisher Scientific	cat#ALF-B22181-0B, CAS: 25322-68-3
Polyethylene glycol 1000	ThermoFisher Scientific	cat#AAB2213430, CAS: 25322-68-3
Polyethylene glycol 4000	ThermoFisher Scientific	cat#ALF-A16151-30, CAS: 25322-68-3
Sodium Chloride	ThermoFisher Scientific	cat#A12313, CAS: 7647-14-5
Glycerol	ThermoFisher Scientific	cat#A16205, CAS: 56-81-5
Naproxen Sodium	TCI chemicals	cat#M3406, CAS: 26159-34-2
Potassium chloride	ThermoFisher Scientific	cat#A11662, CAS: 7447-40-7
Sodium hydrogen phosphate	ThermoFisher Scientific	cat#013437, CAS: 7558-79-4
Potassium dihydrogen phosphate	ThermoFisher Scientific	cat#011594, CAS: 7778-77-0

**Software and algorithms**		

Origin	OriginPro 9.0	https://www.originlab.com/demodownload.aspx
Gromacs	GROmacs 2020.4	https://manual.gromacs.org/documentation/2020.4/download.html
Avogadro	version 1.2.0	https://sourceforge.net/projects/avogadro/files/latest/download
Visual Molecular Dynamics	version 1.9.3	https://www.ks.uiuc.edu/Development/Download/download.cgi?PackageName=VMD

### Method details

#### Fabrication of hydrogel

1.6 g of gelatin was dissolved in 20 mL of water (Model: Milli Q, Millipore Elix water system, resistivity 18 MΩ cm) at 40°C and was allowed to form a homogeneous solution. A full-factorial design of experiments was performed with the required amount of PEG, salt, and glycerol. A sequential addition protocol was followed, where PEG 2000 was first added to the homogeneous solution, followed by PEG 1000, PEG 4000, NaCl, and glycerol, with a gap of 30 min 30 mg of Naproxen sodium (model drug) was added after glycerol to prepare the drug-loaded hydrogel. In the end, 2.4g of sodium alginate was added at a higher stirring speed, which led to physically crosslinked hydrogel preparation. The hydrogels are then frozen for 15 min and dried at 37°C for 72 h.

#### Swelling behavior

The swelling behavior of the physically crosslinked hydrogel was studied in PBS (pH = 7.4) at 37°C. The dried hydrogels were weighed and immersed in PBS, and the swelled weight was measured at different time intervals after removing the excess PBS from the hydrogel surface using tissue paper. The swelling degree (SD) is calculated using the following equation.

(Equation 1) $$SD\;\left(\%\right)=\frac{W_{s}-W_{d}}{W_{d}}\times 100$$

Here, W_s_ and W_d_ denote the weight of the swollen hydrogel and the initial dry weight of the hydrogel, respectively. Further, these values for all the hydrogels were normalized with respect to the highest swelling degree value of the hydrogel, which gives us the Normalized Swelling Degree (NSD). All these experiments have been repeated three times.

#### In-vitro drug release studies

The prepared dried hydrogels were immersed in a 100 mL PBS buffer solution and placed in an incubator shaker at 100 rpm at 37°C. At fixed time intervals, 3mL aliquots were collected, and the drug concentration was analyzed using UV-VIS spectroscopy (Lab India Analytical UV3092). The release factor is calculated using Equation 2.^44^

$$RFN=\frac{Amount\;of\;drug\;released\;\left(mg\right)\;until\;zero\;order\;kinetics}{Total\;amount\;of\;Drug\;loaded\;\left(mg\right)}$$

$$TFN=\frac{Time\;\left(hrs\right)\;until\;which\;zero\;order\;kinetics\;was\;observed}{Total\;time\;of\;drug\;release\;\left(hrs\right)}$$

(Equation 2) $$Release\;Factor\;\left(RF\right)=Release\;Fraction\;\left(RFN\right)\times Time\;Fraction\;\left(TFN\right)\times R^{2}$$

Normalizing the Release Factor value with the highest Release Factor gives the Normalized Release Factor (NRF). All measurements were performed in triplicates.

#### Degradation studies

Once the hydrogels had reached the swelling equilibrium (SD equilibrium), the hydrogel samples were subjected to degradation at 37°C. The weight of the hydrogels was measured at particular time interval. The degradation (%) was calculated as follows:

(Equation 3) $$\text{Degradation}\;\left(\%\right)=\frac{w_{a}}{w_{eq}}\times 100$$

where W_a_ is the weight of hydrogel after reaching equilibrium swelling (at the onset of degradation), and W_eq_ is the weight of hydrogel at equilibrium swelling. These values were plotted with respect to time, and the slope was calculated to depict the degradation rate. These values were normalized with respect to the highest degradation rate and named as Normalized Degradation Rate (NDR). The experiments have been repeated three times.

#### Rheological studies

##### Amplitude sweep test

The amplitude sweep studies were performed at 37°C for the swelled samples to determine linear viscoelastic regime (LVER) using parallel plate geometry (25 mm diameter). The shear strain was taken from 0.01 to 100%. The angular frequency is kept constant at 6.28 rad/s. The storage modulus value obtained at LVER was noted down for all the samples, and each of these values was divided with respect to the highest value, which was named the Normalized Storage Modulus (NSM). All the rheological tests have been repeated three times.

##### Frequency sweep test

The linear viscoelastic regime obtained from the previous test was used to perform frequency sweep studies by varying the angular frequency from 0.01 to 100 Hz. The stability of the hydrogels was determined with time using parallel plate geometry (25 mm diameter). The shear strain is kept constant at 0.1% (linear viscoelastic regime for all hydrogels). The hydrogels are stable if G'> G″ at a lower frequency.

##### Mesh size calculation

The mesh size of the hydrogels was calculated using the rubber elastic theory using the following equation.^68^

(Equation 4) $$\xi ={\left(\frac{G_{e}^{\prime }\;N_{A}}{RT}\right)}^{-\frac{1}{3}}$$

where ξ denotes the average mesh size of the hydrogels, $G_{e}^{\prime }$ is the plateau value of storage modulus obtained from the frequency sweep profiles, N_A_ is Avogadro’s constant, R is the universal gas constant, and T is the temperature. All the mesh size values obtained for the hydrogels were normalized with respect to the highest value, and normalized mesh size (NMS) was collected for the hydrogels.

##### Self-healing behavior

The self-healing properties were evaluated using strain relaxation or alternate step strain measurements. A low strain is given initially (0.1%) within the linear visco-elastic region for 3 min where G'> G" (gel region). Then, the hydrogel structure is destroyed by applying a high strain of 100% for 2 min where G"> G' (sol region). Self-healing behavior is observed on the premise that they can regain back their initial structures. The cycle is repeated three times. The % recovery of the initial structure was calculated using the following equation.^69^

(Equation 5) $$Recovery\;\left(\%\right)=\left(\frac{\;G^{\prime }\left(last\;cycle\right)}{G^{\prime }\left(first\;cycle\right)}\right)\times 100$$

where, $G^{\prime }\left(first\;cycle\right)$ is the storage modulus at the first cycle, i.e., when the hydrogel is at the initial structure at lower strain and $G^{\prime }\left(last\;cycle\right)$ is the storage modulus at the last cycle, i.e., after the hydrogel has recovered from the destructive strain cycles. Similarly, the recovery (%) was normalized with respect to the highest value to acquire Normalized Percentage Recovery (NPR).

#### 1.7 Dynamic Light Scattering (DLS)

1.6 g of gelatin was dissolved in water (20 mL) at 40°C. Once a homogeneous solution was formed, PEG 2000, 1000, 4000, NaCl, and glycerol were added strictly following the hydrogel preparatory method (sequential addition), and the analysis was performed (Figure 1). 3 mL of solution was taken, and the size of gelatin was analyzed using dynamic light scattering (nanoSAQLA, Otsuka).

The CONTIN algorithm was used to process data. The hydrodynamic radius was calculated using the Stokes-Einstein equation (Equation 6).

(Equation 6) $$D=\frac{k_{B}T}{6\pi \eta R_{h}}$$

Where, *D* = Translational Diffusion Coefficient, *k*_*B*_ = Boltzmann Constant, *T* = Temperature, *η* = Viscosity of the solution, *R*_*h*_ = Hydrodynamic Radius. All the measurements were performed in triplicates.

#### Fourier transform infrared spectroscopy/attenuated total reflectance (FTIR/ATR)

The structural modification due to the electrostatic interactions of salt and the H-bond formation by the plasticizers within the polymer matrix and their impact on the hydrogel was studied using the FTIR/ATR technique. The FTIR-ATR analysis was conducted using Bruker Tensor 37, MIRacle Single Reflection Horizontal ATR accessory. The spectral range was 600–4000 cm^−1^ with a spectral resolution of 4 cm^−1^. 256 scans were performed to ensure the reproducibility of the data. Each sample has been repeated three times to check the reproducibility of the data.

#### Molecular dynamic simulation

The molecular dynamics (MD) simulations were performed using the GPU version of the software GROMACS (GROningen Machine for Chemical Simulation, 2020.4).^70^ The atoms in polymers were treated with OPLSAA forcefield with periodic boundary conditions in all three dimensions while TIP4P water model was chosen for the water molecules.^71^ These parameters were chosen as they give much more realistic results when compared with experiment (for liquid water).^72^ The initial configurations were prepared using Avogadro (version 1.2.0) software.^73^ In this software, SMILES (Simplified Molecular Input Line Entry System) text was used as an input to generate the molecular structure. The GROMACS topology was generated using TopolGen (version 1.1). This script provides an initial topology file that requires modifications. The modifications were done by building script files for each polymer molecule. VMD (Visual Molecular Dynamics, version 1.9.3) software was used for visualizing the molecular structure.^74^ Energy minimization was performed for each system using the steepest descent algorithm until a maximum force of less than 1000 kJ/mol/nm was reached. This was done to avoid any steric repulsions in the system. A 5 ns NVT simulation was conducted with the energy minimized structure, followed by a 20 ns MD production. The leapfrog algorithm was used to integrate the equation of motion and the time step used as 1fs. LINCS algorithm was employed to constrain all the bonds.^75^ The system temperature was maintained at 310 K using the Nosé-Hover thermostat.^76^ The long-range electrostatic interactions were computed using the particle mesh Ewald (PME) method.^77^ The cut-off distance for van der Waals and the short-range electrostatic interactions were taken as 1 nm. The simulation trajectory of the MD production run for the last 1000 time frames separated by 5 ps was used to calculate the average over all the statistics. Five gelatin chains of molecular weight 20000 g/mol were used. The box length was chosen equivalent to the experimental 8% w/v concentration i.e., 12.75 nm. To maintain the sequential addition protocol of the experiments, PEG 2K, PEG 1K, PEG 4K, NaCl, and glycerol were added one by one to the final simulated state of each case.

### Quantification and statistical analysis

All the data are presented as the mean value ±standard deviation using OriginPro 9 from the experiments performed at least three times. Statistical analysis was carried out by one-way analysis of variance (ANOVA) to determine whether differences between the test groups were statistically significant with 95% confidence (*p* < 0.05) (Table S4). The minimum number of replicates was three for each test, represented as ‘n’ values, which can also be found in figure legends. The analysis showed that plasticizers and salt have an effect on the hydrogel properties.
