# Supplementary material for: Fabrication of crosslinker free hydrogels with diverse properties: An interplay of multiscale physical forces within polymer matrix
Source: iScience. 2024 Oct 22;27(11):111227. doi: 10.1016/j.isci.2024.111227 (PMC11574810; doi:10.1016/j.isci.2024.111227)
Supplement: Document S1. Figures S1–S3 and Tables S1–S4 [file mmc1.pdf]

iScience, Volume *27*

Supplemental information

Fabrication of crosslinker free hydrogels with diverse properties: An interplay of multiscale physical forces within polymer matrix

Tithi Basu, Debasish Goswami, and Saptarshi Majumdar

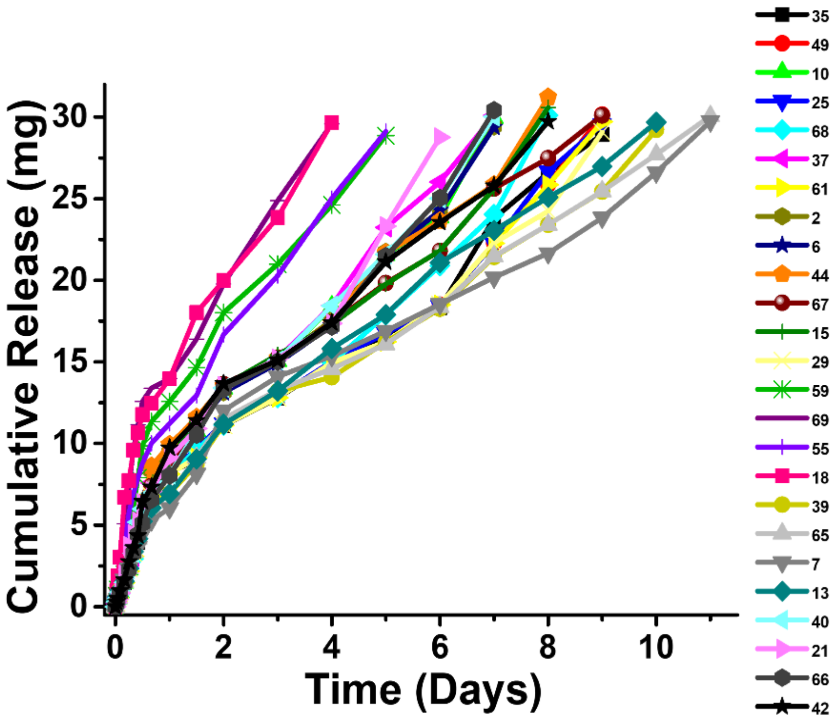

Figure S1. Cumulative release for representative samples. The release fraction was calculated from the plot till zero-order release was observed. Data presented here is representative of curve obtained from n=3 samples. Related to Results and Discussions, section 2.2.

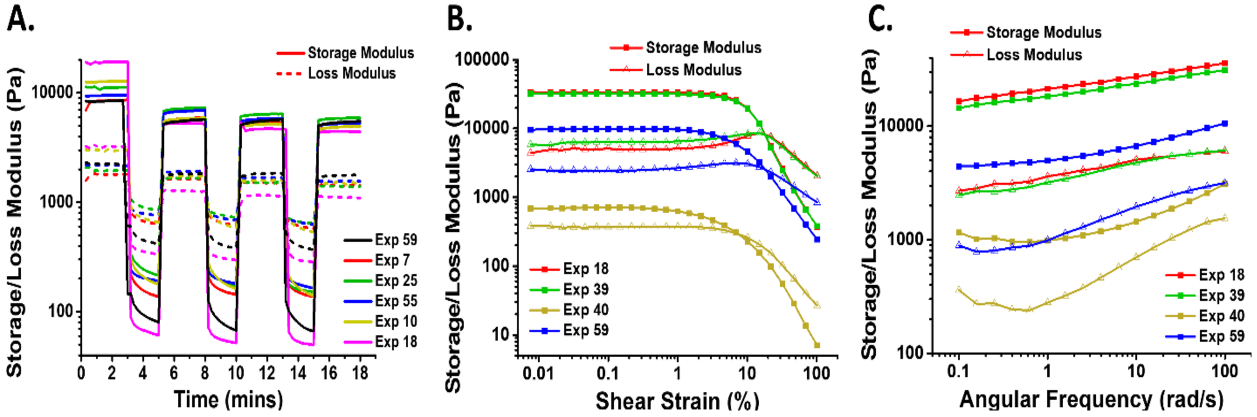

Figure S2. Rheology analysis. Data presented here is representative of curve obtained from n=3 samples. A) Self-healing: alternate step strain measurement of the samples with respect to time, Exp 59 showing higher recovery; B) Storage and Loss modulus values from amplitude sweep showing Exp 18 and 39 with highest storage modulus; C) Storage and Loss modulus values from frequency sweep showing storage modulus higher than loss modulus. Related to Results and Discussions, section 2.3 & 2.5.

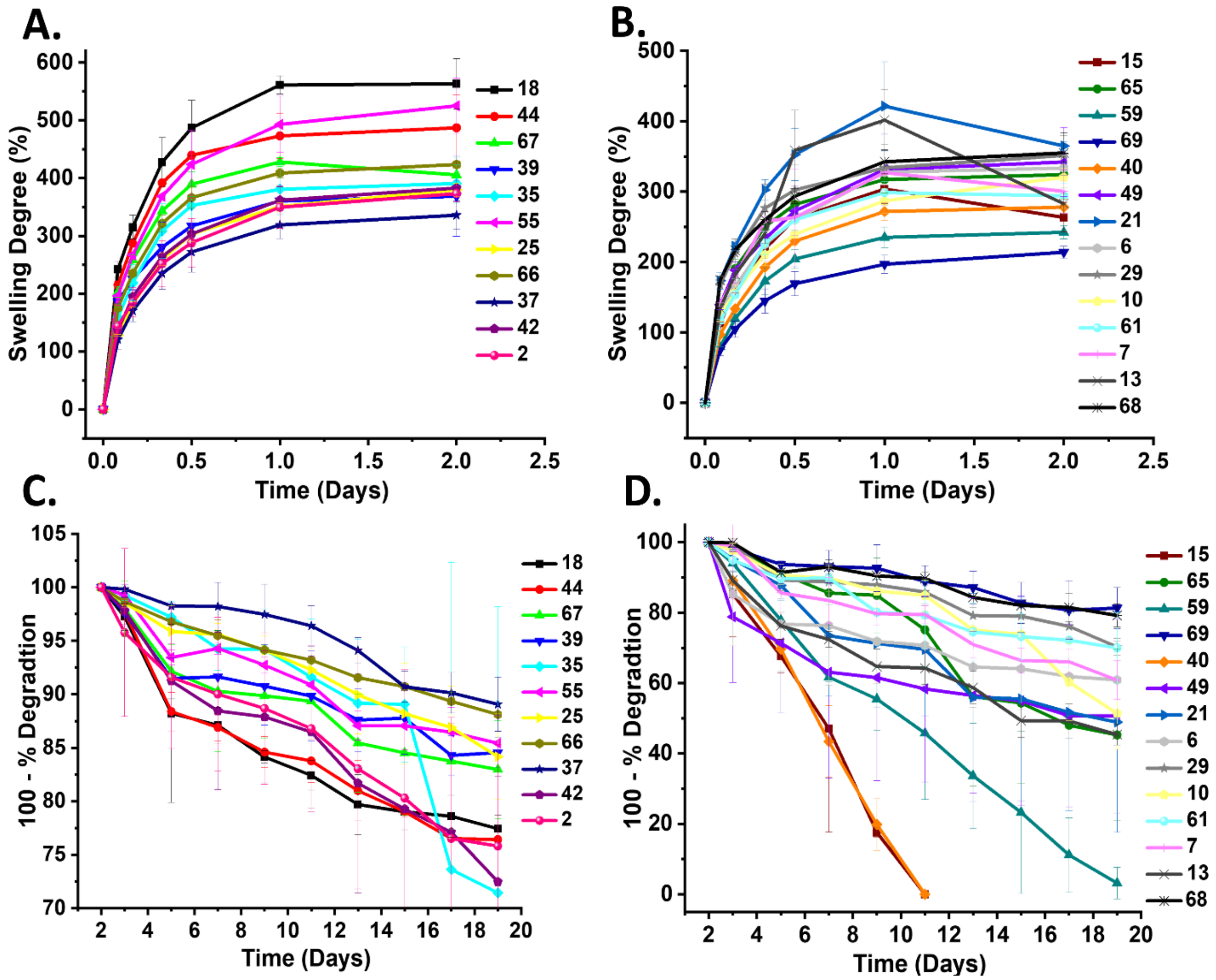

Figure S3. Swelling degree over the time till 2 days for representative samples: A), B). Data are represented as mean ± SD obtained from n = 3 samples. Percentage degradation over the time (from 2nd day) for representative samples: C), D). Data are represented as mean ± SD obtained from n = 3 samples. The degradation rate was calculated from the slope of the curve. Related to Results and Discussions, section 2.6.

Table S1. Total set of experiments. Related to Figure 3.

Exp	PEG 2000 (g)	PEG 1000 (g)	PEG 4000 (g)	NaCl (mg)	Glycerol (mL)
1	0.5	0	0	0	1.5
2	0.5	0.75	0.75	0	0
3	0.5	0	0.75	0	0
4	0	0.75	0	0	0
5	1	0.75	0.75	0	1.5
6	0	0.75	0.75	75	0
7	0.5	0.75	0	150	1.5
8	0	0.75	0	150	0
9	0	0	0.75	150	1.5
10	0.5	0.75	0.75	75	0
11	1	0	0	0	1.5
12	1	0	0	75	0
13	0.5	0	0.75	150	0
14	0.5	0.75	0	0	1.5
15	0	0.75	0.75	150	0
16	0	0	0	75	1.5
17	0.5	0	0.75	0	1.5
18	0	0	0	0	0
19	1	0	0	0	0
20	1	0	0.75	150	1.5
21	1	0	0	150	0
22	1	0	0.75	75	1.5
23	0	0	0	150	0
24	0	0	0.75	150	0
25	0	0	0.75	0	1.5
26	0	0.75	0	75	1.5
27	1	0.75	0.75	75	0
28	0.5	0	0.75	75	0
29	0	0.75	0	150	1.5
30	1	0	0.75	75	0
31	0	0.75	0.75	0	1.5
32	1	0.75	0	150	1.5
33	1	0	0.75	150	0
34	1	0	0.75	0	0
35	0	0.75	0	0	1.5
36	0.5	0.75	0.75	0	1.5
37	1	0.75	0	0	0
38	0.5	0	0	150	1.5
39	0.5	0	0	75	0

40	1	0.75	0.75	150	0
41	0	0.75	0.75	75	1.5
42	0.5	0.75	0	75	0
43	1	0.75	0	0	1.5
44	0.5	0	0	0	0
45	1	0	0	75	1.5
46	0.5	0	0	150	0
47	1	0.75	0.75	0	0
48	0	0.75	0.75	150	1.5
49	0.5	0.75	0	75	1.5
50	0.5	0.75	0.75	150	1.5
51	0.5	0	0	75	1.5
52	0.5	0.75	0.75	75	1.5
53	1	0	0.75	0	1.5
54	1	0.75	0	150	0
55	0	0	0	0	1.5
56	0	0	0.75	0	0
57	0.5	0.75	0	0	0
58	1	0.75	0	75	1.5
59	1	0.75	0.75	150	1.5
60	1	0.75	0	75	0
61	0.5	0	0.75	150	1.5
62	0.5	0.75	0.75	150	0
63	0	0.75	0.75	0	0
64	0	0	0.75	75	1.5
65	1	0	0	150	1.5
66	0	0.75	0	75	0
67	0	0	0	75	0
68	0.5	0.75	0	150	0
69	1	0.75	0.75	75	1.5
70	0	0	0.75	75	0
71	0.5	0	0.75	75	1.5
72	0	0	0	150	1.5

Table S2. Representative experimental points from clusters. Related to Figure 3.

Exp	PEG 2000 (g)	PEG 1000 (g)	PEG 4000 (g)	NaCl (mg)	Glycerol (mL)
59	1	0.75	0.75	150	1.5
69	1	0.75	0.75	75	1.5
40	1	0.75	0.75	150	0
49	0.5	0.75	0	75	1.5
2	0.5	0.75	0.75	0	0
21	1	0	0	150	0
6	0	0.75	0.75	75	0
29	0	0.75	0	150	1.5
15	0	0.75	0.75	150	0
10	0.5	0.75	0.75	75	0
61	0.5	0	0.75	150	1.5
65	1	0	0	150	1.5
7	0.5	0.75	0	150	1.5
13	0.5	0	0.75	150	0
66	0	0.75	0	75	0
37	1	0.75	0	0	0
42	0.5	0.75	0	75	0
68	0.5	0.75	0	150	0
39	0.5	0	0	75	0
35	0	0.75	0	0	1.5
55	0	0	0	0	1.5
25	0	0	0.75	0	1.5
18	0	0	0	0	0
67	0	0	0	75	0
44	0.5	0	0	0	0

Table S3. Percentage recovery, mesh size and storage modulus from representative points. Related to Figure 6 and 7.

Exp	Percentage recovery (%)	Mesh size (nm)	Storage modulus (G') (Pa)
18	23.2	6.09	33418
44	36.84	6.52	21689
67	33.35	5.97	23789
39	19	6.38	31992
35	52.4	7.78	15803
55	55.02	7.09	24969
25	55.14	8.33	13417
66	41.41	6.1	27321
37	35.15	6.27	21064
42	18.34	6.37	27574
15	52.81	8	12685
65	37.66	8.04	20431
59	63.32	9.71	9885.7
69	55.3	8.58	1153
40	57.48	16.1	704.51
49	56.53	6.78	13913
2	33	6.28	23168
21	30.64	6.56	12377
6	41.34	6.71	14742
29	42.79	6.49	13315
10	30.1	8.35	16690
61	36.5	8.79	11940
7	56.83	8.15	16356
13	38.06	8.12	17353
68	36.5	6.22	15040

Table S4. Statistical analysis on the data for effect of plasticizers and salt (components) on the swelling degree, gel strength, drug release, percentage recovery, mesh size, storage modulus, degradation of the hydrogel. Related to STAR Methods, QUANTIFICATION AND STATISTICAL ANALYSIS.

Case	F Value	F critical	P-value	NULL Hypothesis	Result
Effect of components on swelling degree	15.04	1.96	1.29E-09	There is no effect of components on the swelling degree.	F value > F critical and p value < 0.05 => NULL Hypothesis rejected.
Effect of components on gel strength	48.99	1.96	1.43E-15	There is no effect of components on the gel strength.	F value > F critical and p value < 0.05 => NULL Hypothesis rejected.
Effect of components on drug release	33.87	1.96	1.17E-13	There is no effect of components on the drug release	F value > F critical and p value < 0.05 => NULL Hypothesis rejected.
Effect of components on percentage recovery	38.09	1.96	2.89E-14	There is no effect of components on the percentage recovery	F value > F critical and p value < 0.05 => NULL Hypothesis rejected.
Effect of components on mesh size	49.74	1.96	1.19E-15	There is no effect of components on the mesh size	F value > F critical and p value < 0.05 => NULL Hypothesis rejected.
Effect of components on storage modulus	68.23	1.96	2.59E-17	There is no effect of components on the storage modulus	F value > F critical and p value < 0.05 => NULL Hypothesis rejected.
Effect of components on degradation	17.42	1.96	2.54E-10	There is no effect of components on the degradation	F value > F critical and p value < 0.05 => NULL Hypothesis rejected.
